# How to maintain the maximal level of blinding in randomisation for a placebo-controlled drug trial

**DOI:** 10.1016/j.conctc.2019.100356

**Published:** 2019-04-09

**Authors:** Lei Clifton, David A. Clifton

**Affiliations:** a Nuffield Department of Population Health (NDPH), University of Oxford, UK; b Institute of Biomedical Engineering (IBME), Department of Engineering Science, University of Oxford, UK

**Keywords:** Randomisation, Blinding, Single-blind, Double-blind, Placebo, Randomised controlled trial (RCT), Investigational medical product (IMP), Pharmacist

## Abstract

We illustrate the approach of randomising treatments and compare it with the traditional approach of randomising patients, using a case study drawn from the authors’ experience in clinical trials. The setting is a double-blind parallel two-arm randomised controlled trial (RCT), but the method in this paper can be extended to single-blind, cross-over, or multi-arm RCTs.

We propose the concept of two different levels of blinding: full blinding and partial blinding. We subsequently show how to maintain the maximal level of blinding. Using an example, we show that a pharmacist can be fully blinded if the investigational medical products (IMPs) that they prescribe (instead of patients) are randomised, and they can be partially blinded if they need to dispense replacement (i.e., surplus) IMPs. A small number of surplus IMPs is commonly required in a clinical trial to replace lost or damaged IMPs. We note that the concept of full blinding and partial blinding is different from double-blind trial, and the level of blinding is relevant in both single-blind and double-blind trials.

A trial statistician needs to work closely with all parties in the design of the randomisation, including the pharmacist, the trial manager, and the manufacturer. We detail what should and should not be shown in the various documents that the trial statistician need to provide to the pharmacist and to the manufacturer. We provide template tables for these documents.

## 1 Background

The importance of blinding in a randomised controlled trial (RCT) is widely accepted [[1], [2], [3], [4], [5]], but there has been little practical guidance on how blinding is achieved and maintained during the implementation of randomisation. Accomplishing successful randomisation and blinding requires the trial statistician, the trial manager, the pharmacist, the principle investigator (PI), and the manufacturer to work closely together. The pharmacist is responsible for dispensing and recording the allocated investigational medical products (IMPs) to patients, following the randomisation list provided by the trial statistician. The trial manager coordinates the process of randomisation. The PI is blinded throughout the trial, but they are involved in designing the randomisation and blinding. The trial statistician needs to provide separate documents to the manufacturer and the pharmacy to describe the randomisation process.

It is important to note that blinding and randomisation are two different aspects of an RCT. Random treatment allocation can always be achieved in an RCT regardless of whether the trial is open label, single-blind, or double-blind.

The subtlety of different levels of blinding may go unconsidered when implementing randomisation in an RCT. In this paper, we introduce the concept of two levels of blinding, using an example drawn from the authors’ experiences in medical statistics for clinical trials.

## 2 Method

### 2.1 Two levels of blinding

The setting for our case study is a parallel two-arm RCT, assessing an IMP that is dispensed in vials by the pharmacist. The RCT is double-blind, in which both the clinician and the patients are blinded to the actual IMP allocated to the patient; i.e., neither the clinician nor the patient know whether the patient has received active drug or placebo. The concept of different levels of blinding we introduced in this paper is relevant in both single-blind and double-blind trials. The method is also applicable for crossover and multi-arm RCTs.

There are 40 patients in total, of whom 20 are assigned to the active drug and 20 to the matching placebo. Each patient is allocated 10 vials of the same IMP (either active drug or placebo) during the course of the trial; therefore a total of 400 vials (called “planned vials” in this paper) are required. A small number of surplus vials also need to be manufactured to replace damaged or missing vials, and it is deemed that 60 surplus vials are sufficient. The IMP is manufactured in two batches, due to its shelf life. The pharmacist will dispense the IMP in vials.

In this paper, we describe two levels of blinding of the pharmacist:

Full blinding. The pharmacist has no knowledge of whether the allocation IMP to a patient is the active drug or the placebo. The pharmacist simply dispenses the IMP following a randomisation list provided by the statistician, shown in Table 3. All vials have identical appearance with no treatment allocation displayed. Each vial has a unique serial number that the pharmacist uses for assigning the allocated treatment to each patient.
Partial blinding. The pharmacist will see that some patients receive the same IMP, but they do not know whether the IMP is active drug or placebo. We show that the pharmacist can still be partially blinded when replacement vials need to be dispensed to a patient; an example is shown in Table 5.

In the approach described in this paper, the pharmacist is fully blinded when dispensing the planned vials, and will become partially blinded when surplus vials are needed. Patients are fully blinded, and will remain fully blinded even if they need surplus vials. The clinician is fully blinded, but both the pharmacist and the clinician will be unblinded in the event of emergency unblinding.

To our knowledge, there is no previous work on how to maintain maximal blinding when the randomisation unit is treatments (i.e., vials in the example in this paper). We propose the novel concept of full blinding and partial blinding in this paper, and describe how to achieve them using an example derived from real world experience.

### 2.2 Randomising patients vs. randomising treatments

The most common and intuitive randomisation approach is randomising patients (rather than randomising treatments). The statistician gives the pharmacist the randomisation list. If patients (instead of vials) are randomised, the manufacturer will produce vials with a layer of removable labelling that show whether the IMP is active drug or placebo. The pharmacist will remove this layer of label before dispensing the vial to the patient. The pharmacist is not blinded, but the patients and the clinician are blinded.

The above approach will work if the labels on the vials are removable; however, this may not always be feasible. In the example in this paper, the vials must have permanent labels from the manufacturing process due to regulatory requirements. In this instance, one has to randomise the vials instead of patients.

Randomising treatments is also commonly used in ambulance-based RCTs, where randomising patients is not feasible. One example is the RIGHT trial [6], in which each paramedic carries an opaque sealed envelope containing the treatment allocation; this envelope is only opened if informed consent is obtained. Another example is the PARAMEDIC-2 trial in which each ambulance carries the randomised trial treatment packs that contain either adrenaline or placebo [7].

Randomising patients allows the randomisation to be stratified by patient characteristics, such as gender, age group, etc. The most commonly used randomisation methods are stratified permuted blocks [8] and minimisation [9].

By contrast, randomising treatments does not involve any patient characteristic, and therefore cannot be stratified by patient characteristics or using minimisation. The random treatment allocation is usually pre-determined. Randomisation can be achieved by simple randomisation or permuted blocks.

Table 1 compares the two randomisation approaches in the context of the example in this paper. The pharmacist will be unblinded if patients are randomised, but they can be blinded if vials are randomised. In the following sections, we describe how blinding of the pharmacist can be achieved and maintained when the vials are randomised.

**Table 1:** Comparing two different randomisation approaches: randomising patients vs. randomising vials.

	Randomising patients	Randomising vials
Labels on the vial	Two layer of labels: the top layer must be removable; the bottom layer must not reveal the actual IMP.	One layer of permanent label. This label must not reveal the actual IMP.
Blinding of the pharmacist	Not blinded	Blinded
Manufacturer	Removable labels on vials.	Permanent (i.e. non-removable) labels on vials.
Content of vial (i.e. active or placebo) after dispensing to patients	Not identifiable^a^ once the top layer of the label is removed	Identifiable by its unique number^b^ printed on the label
Randomisation method	Can be stratification or minimisation.	Pre-determined; cannot be stratified by any patient characteristics.

a If the manufacturer prints a unique number on each vial and keeps a record of that number and its corresponding IMP, the content in the vial can still be identified.

b This unique number is the “vial number” in the example in this paper; the statistician provides the manufacturer with a table that matches the vial number with its corresponding IMP, shown in Table 3.

In our example, the vials must have permanent labels during manufacturing; therefore, random treatment allocation has to be achieved by randomising vials instead of patients. Each vial has its own unique serial number printed on its label.

## 3 Tables provided to different parties for different purposes

### 3.1 What to print on the label of the vial

During randomisation, each patient is assigned a randomisation number sequentially; the first patient at the pharmacist is given randomisation number 01, the second patient is given randomisation number 02, and the last patient will have randomisation number 40. We note that the randomisation number is different from the patient ID. A patient is usually assigned a patient ID before randomisation during the screening or recruitment stage. The randomisation number is different from the patient ID; they are both necessary in an RCT. The pharmacist will assign the randomisation number sequentially to each patient ID, and therefore each randomisation number is matched to a patient ID via the table held by the pharmacist.

The trial team is allowed to specify what printing shall appear on the labels. The trial statistician is essential in specifying what shall be printed on the label of the vials, shown in Table 2 below.

**Table 2:** Printing on the permanent (i.e. non-removable) label of the vial.

Trial Name	Example Trial
IMP	Active/Placebo
Randomisation Number^a^	01
Vial Number^b^	1001

a The range of randomisation number is 01–40. This randomisation number is different from the patient ID, as explained in the main text.

b The range of vial number is 1001–1230 for the 1st batch, and 2001–2230 for the 2nd batch.

We note that one must not print “Treatment A″ or “Treatment B″ on the vials, because this will compromise the blinding. The clinician, the pharmacist, and the patients can all see that patients whose vial shows “Treatment A″ has received a different treatment from patients whose vial shows “Treatment B”. The unblinding of one patient's treatment would therefore reveal the treatment allocation of all patients in the trial.

### 3.2 Table to the manufacturer

The statistician provides the manufacturer with a table containing the following information on the vial number and treatment allocation to the IMP. Vials are allocated at random to either the active IMP or placebo, because the randomisation unit is vials. The manufacturer receives the matching between the *vial* number and an IMP, but not the matching between *randomisation* number with an IMP, because the manufacturer is not involved in the randomisation process. The pharmacist must not see Table 3, because it will unblind them for the planned vials. The next section will show that the pharmacist will have to be unblinded while dispensing the surplus vials.

Table 3 shows the total 460 vials that are required (400 planned and 60 surplus vials), but it should not specify which ones are planned vials and which ones are surplus vials, because such information is irrelevant to the manufacturing process. In two separate tables to the pharmacy, the trial statistician will specify which vials are to be used for the planned vials (Table 4) and surplus vials (Table 5), respectively. The vials shown on Table 3 will need to match the tables (Table 4, Table 5) to the pharmacist; it is the responsibility of the trail statistician to ensure all tables contain consistent information. The trial statistician should ensure and check the matching before delivering different tables to relevant parties.

**Table 3:** Template for the table to the manufacturer, There are 460 vials in total, produced in two batches. The 230 vials in each batch has an equal number of active drugs and placebos. In this example, the trial statistician has decided that in each batch, the first 200 vials will be planned vials, while the remaining 30 vials will be surplus vials. This sequence can be changed.

Batch number	Vial number range	IMP
1	1001–1100	Active
	1101–1200	Placebo
	1201–1215	Active
	1216–1230	Placebo
2	2001–2100	Active
	2101–2200	Placebo
	2201–2215	Active
	2216–2230	Placebo

**Table 4:** Table to the pharmacist, prepared by the trial statistician, showing the planned 400 vials for the 40 patients in total, produced in two separate batches. The pharmacist is blinded to the treatment allocation of the IMP for the planned vials.

Batch number	Randomisation Number	Vial number range^a^
1	01	1021–1030
	02	1191–1200
	…	…
	20	1081–1090
2	21	2071–2080
	22	2011–2020
	…	…
	40	2131–2140

a The total range of planned vial numbers is 1001–1200 in the 1st batch, and is 2001–2200 in the 2nd batch. Each patient is randomly assigned 10 vials in consecutive numbers.

**Table 5:** Table to the pharmacist, prepared by the trial statistician, showing the 60 surplus vials in total, together with allocated IMP. The 30 surplus vials in each batch is a mixture of active drug and placebo. The pharmacist will become partially blinded to the treatment allocation of the IMP when dispensing the surplus vials.

Batch Number	Randomisation Number	Surplus vial numbers^a^
1	01	1216–1230
	02	1201–1215
	…	…
	20	1201–1215
2	21	2216–2230
	22	2201–2215
	…	…
	40	2216–2230

a The total range of surplus vial numbers is 1201–1230 in the 1st batch, and is 2201–2230 in the 2nd batch. The vials in the 1st batch are assigned into two blocks: 1201–1215, and 1216–1230. Similarly, the vials in the 2nd batch are assigned into blocks: 2201–2215, and 2216–2230. The pharmacists do not know which of two blocks is for placebo or active drug, although they can see the two blocks in each batch.

As noted earlier, the first digit of the vial number indicates the batch number: 1 for the first batch, and 2 for the second batch. The remaining three digits denote the vial number in a specific batch.

### 3.3 Table to the pharmacist for the planned vials

The statistician provides Table 4 to the pharmacist, in which each patient is randomly assigned 10 vials in consecutive numbers. Patients in the same batch have the same surplus vial range, because it is not predictable who will need replacement vials and in what quantity. The surplus stock in each batch will be a mixture of both active drug and placebo, and our solution is to provide a separate table (Table 5) to the pharmacy for dispensing the surplus stock. We have decided not to specify any surplus vial range in Table 4 to avoid confusion when assigning (planned or surplus) vials to a patient. We note that Table 4 must not show vial numbers of the surplus vials, because this will compromise the full blinding of the pharmacist.

### 3.4 Table to the pharmacist for the surplus vials

The use of surplus vials requires careful consideration. The trial team has decided that a total of 60 surplus vials is needed, with 30 to be manufactured in the first batch (vial numbers 1201–1230) and 30 in the second batch (vial numbers 2201–2230). The pharmacist will need to ensure that the patient receives the same replacement IMP as assigned, but the difficulty is that the pharmacist is fully blinded to the treatment allocation. Our solution is to provide the pharmacist with a separate table showing the surplus vials for each patient. This table should only be used if replacement vials from the surplus stock need to be dispensed. A template is shown in Table 5 below.

In Table 5, all patients in the 1st batch whose treatment allocation is placebo will have the surplus vial numbers of 1216–1230, while those on active drug will have 1201–1215. The pharmacist is not fully blinded any more, but the maximal possible level of blinding is still achieved. For example, the pharmacist can see that randomisation number 02 and 20 have the same surplus vial numbers, indicating that they have been allocated to the same IMP, but the pharmacist does not know whether the IMP is active drug or placebo. The pharmacist will be fully blinded (i.e., the 1st level of blinding in Section 2) if surplus vials are not needed, and will become partially blinded (i.e., the 2nd level of blinding shown in Section 2) after dispensing a surplus vial to a patient.

### 3.5 Table to the pharmacy for unblinding

Unblinding is a different matter from the consideration of the surplus vials described above. The treatment allocation is revealed during unblinding, while dispensing surplus vials does not require the treatment allocation to be revealed. Unblinding should be avoid at all costs in an RCT; however, emergency unblinding may be required in a trial, for example, when the knowledge of the administered medication alters important clinical decision. The trial protocol should specify when emergency unblinding should take place, and how it should be achieved.

Our solution is to provide the pharmacy with a separate table to be used only for the purpose of unblinding. This table should be kept in a secure location, and only accessed by a pharmacist when unblinding is needed; if possible, this pharmacist should be a different pharmacist from the one who regularly dispenses the vials. A template is shown in Table 6 below. We note that Table 6 should only show the treatment allocation, not the vial numbers of the surplus vials. The surplus vials should be provided to the pharmacy in a separate table (Table 5), as discussed in the previous section.

**Table 6:** Table to the pharmacist, prepared by the trial statistician, showing treatment allocation of the IMP. This table will unblind the pharmacist, and should only be used in the event of emergency unblinding as per the trial protocol.

Batch Number	Randomisation Number	IMP
1	01	Placebo
	02	Active
	…	…
	20	Active
2	21	Placebo
	22	Active
	…	…
	40	Placebo

## 4 Discussion

### 4.1 Applying partial blinding when patients are randomised

We can apply the method of partial blinding to maximise the blinding of the pharmacists when patients (instead treatments) are randomised. We have stated in Section 2.2 that when the randomisation unit is patients, the usual approach is that the manufacture will provide vials with a layer of removable label showing whether the IMP is active drug or placebo, and hence the pharmacist is not blinded. Using the method of partial blinding introduced in this paper, the statistician can provide a table to the manufacturer with label “A” corresponding to active drug and label “B” corresponding to placebo, and prepare another table to the pharmacist indicating who are to take treatment “A” and “B”, respectively. This way the pharmacist may only know which patients receive the same treatments, but without knowing the actual treatment type. This would lead to the partial blinding of pharmacist as defined in Section 2.1.

### 4.2 An alternative approach for the surplus vials

In Sections 3.3 and 3.4, we outline the situation when the pharmacist have to reveal the table for the surplus stock, which would compromise the full blinding of the pharmacist because the same vial number range would correspond to the same type of treatment. A potential alternative is to have surplus vials assigned to each patient from the beginning of the study.

For example, each patient will have 11 instead of 10 vials assigned in Table 4, and then there are 10 surplus vials left to be assigned in Table 5. In the case when an extra vial is needed, the pharmacist could follow the original assignment in Table 4 to assign one surplus vial and follow Table 5 if more than one surplus vial is needed for the same patient. In such a case, unless more than one vial is needed for the same patient, the full blinding of the pharmacist is maintained.

This approach increases the probability of the full blinding of the pharmacist but is less flexible than the original approach. The extra single vial assigned to each patient may lead to potential wastage. However, this alternative approach may be desirable according to the needs of the study, and therefore has its place in trial design.

### 4.3 Matching randomisation number with patient ID

The pharmacy typically has its own table for recording which randomisation number is assigned to which patient. At the minimum, the table should contain a patient identifier (e.g., patient ID or name) and its corresponding randomisation number. The pharmacy also typically records the date of dispensing along with the signature of the pharmacist on the table. The trial statistician is not involved in this record-keeping due to patient confidentiality, although they will receive the matching between the randomisation number and the patient ID at the data analysis stage, after the patient data have been anonymised.

### 4.4 Engaging the pharmacy early

The pharmacy is a crucial part for dispensing the allocated IMPs to patients, and their records also allow each patient to be matched to their received IMPs. The pharmacy may also need funding to support the trial, for the storage of the IMPs and for their staff time. In this example, the pharmacy will have to store all the 230 vials produced in each batch, and will have to consider the available shelf or fridge space. It is important to engage the pharmacy at the design stage of the trial, to ensure their requirements are met and their costs are included in the funding application.

### 4.5 Limitations

Although the method in this paper can be extended to randomisations when the randomisation list can be pre-specified, it is not applicable when the randomisation sequence is not pre-determined.

The different levels of blinding in this paper are only relevant when blinding the pharmacists is feasible in the first place. Randomising treatments (as opposed to patients) allows the pharmacists to be blinded. In contrast, if patients (instead of treatments) are randomised, the pharmacists will have to be unblinded, and therefore the two different levels of blinding for the pharmacist will not exist.

We did not consider the implications of randomisation errors on blinding, but a guidance on what to do when randomisation goes wrong can be found in Ref. [10].

## Ethics approval and consent to participate

N/A. Not required.

## Consent for publication

Yes.

## Availability of data and material

N/A. Not required.

## Competing interests

None.

## Funding

N/A.

## Authors' contributions

LC conceived the research idea, and led the writing of the paper. DC contributed to writing the paper.

## Acknowledgements

N/A.
